# How to Heal the Gut’s Brain: Regeneration of the Enteric Nervous System

**DOI:** 10.3390/ijms23094799

**Published:** 2022-04-27

**Authors:** Helen Rueckert, Julia Ganz

**Affiliations:** 1 Department of Integrative Biology, Michigan State University, East Lansing, MI 48824, USA; helen.rueckert@duke.edu; 2 Department of Cell Biology, Duke University School of Medicine, Durham, NC 27710, USA; 3 Department of Orthopaedic Surgery, Duke University School of Medicine, Durham, NC 27710, USA

**Keywords:** enteric progenitor cell, zebrafish, inflammation, Hirschsprung disease, neural crest cell, ENS neuropathies

## Abstract

The neural-crest-derived enteric nervous system (ENS) is the intrinsic nervous system of the gastrointestinal (GI) tract and controls all gut functions, including motility. Lack of ENS neurons causes various ENS disorders such as Hirschsprung Disease. One treatment option for ENS disorders includes the activation of resident stem cells to regenerate ENS neurons. Regeneration in the ENS has mainly been studied in mammalian species using surgical or chemically induced injury methods. These mammalian studies showed a variety of regenerative responses with generally limited regeneration of ENS neurons but (partial) regrowth and functional recovery of nerve fibers. Several aspects might contribute to the variety in regenerative responses, including observation time after injury, species, and gut region targeted. Zebrafish have recently emerged as a promising model system to study ENS regeneration as larvae possess the ability to generate new neurons after ablation. As the next steps in ENS regeneration research, we need a detailed understanding of how regeneration is regulated on a cellular and molecular level in animal models with both high and low regenerative capacity. Understanding the regulatory programs necessary for robust ENS regeneration will pave the way for using neural regeneration as a therapeutic approach to treating ENS disorders.

## 1. Introduction

**Figure 1:**
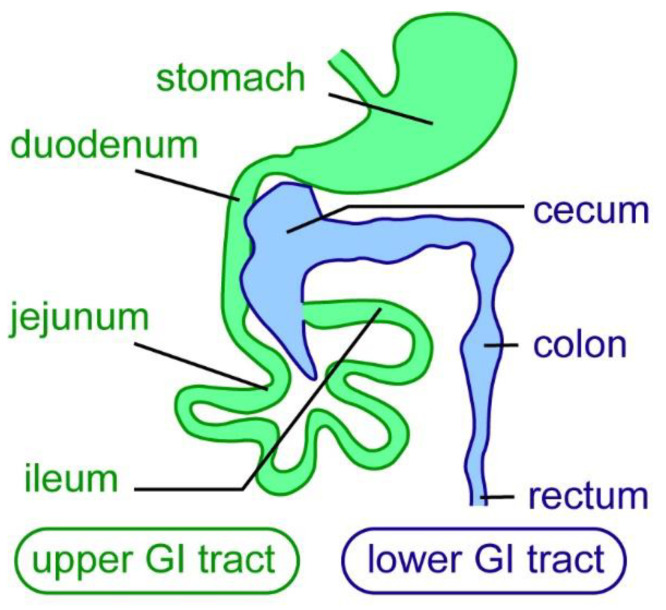
Subdivisions of the mammalian gastrointestinal tract. In mammals the gastrointestinal (GI) tract, here exemplified in mice, is divided into an upper (green) and a lower GI tract (blue). The upper GI tract consist of duodenum, jejunum, and ileum. The lower GI tract consists of the cecum, colon, and rectum [10].

The enteric nervous system (ENS) is derived from the neural crest cell lineage and provides the intrinsic innervation of the gastrointestinal (GI) tract, forming a complex network of different types of neurons and glial cells [1,2,3]. It is the largest division of the peripheral nervous system—the ENS contains approximately the same number of neurons as the adult spinal cord [4,5,6]. The mammalian ENS consists of two enteric plexi: the myenteric plexus, and the submucosal plexus. ENS neurons are found in ganglia, which are connected by a network of nerve fibers [5]. As the intrinsic nervous system of the gut, the ENS regulates many essential intestinal functions, such as GI motility, absorption of nutrients, secretion, fluid exchange, regulation of blood flow, epithelial barrier function, immune modulation, and microbiota colonization and composition [5,7,8,9]. The ENS regulates intestinal functions along the entire length of the gut in mammals; the GI tract can be subdivided into upper and lower parts. The upper GI tract contains the stomach and small intestine (duodenum, jejunum, and ileum), and the lower GI tract contains the cecum, colon, and rectum (Figure 1).

Each of these gut regions is exposed to daily abrasive and potentially harmful and toxic compounds, which in turn subject the ENS to many stressors and mechanical forces. As the ENS directly overlays the intestinal smooth muscle layer, it changes its shape during the gastrointestinal contractions and relaxations that accompany intestinal motility [11]. In aging animals, ENS neuron numbers are considerably reduced [11,12,13]. In addition, acute and chronic gut disorders can also impact ENS cells. Deficits in ENS neuron abundance and composition cause severe GI dysfunction that occur in congenital ENS disorders, such as Hirschsprung disease; inflammatory gut diseases, such as inflammatory bowel syndrome; and neurodegenerative diseases, such as Parkinson’s Disease [14,15,16,17,18,19]. ENS defects in these various disorders range from a complete deficit of neurons in a gut subdivision to a lack of specific neuronal subtypes. For example, in Hirschsprung Disease, ENS neurons are lacking in the distal gut, resulting in difficulties in passing stool. The length of the aganglionic part varies from the distal colon to the entire colon and stretches of the small intestine [15].

## 2. Therapeutic Approaches to Treat ENS Disorders

At present, ENS disorders are treated symptomatically or require surgical removal of the area with ENS neuron deficits [15,18,20,21]. Because of the prevalence of ENS disorders and their strong impact on the patient’s quality of life, there has been an increasing interest in finding therapeutic approaches for restoring lost ENS neurons or glial cells.

There are two main avenues for treating such ENS disorders: ENS stem cell-based treatment or stimulation of resident stem cells to regenerate missing ENS cells [20].

Stem-cell-based therapeutic approaches aim to transplant ENS stem cells into a patient’s gut where they then can differentiate into ENS neurons and/or ENS glial cells [20,22]. In mammals, including humans, enteric neuronal stem cell (ENSC) populations are present into adulthood [22,23]. When isolated and induced in a culture, ENSCs can be amplified and differentiated into many enteric neuronal and glial subtypes [22,24]. Additionally, recent work has generated different types of ENS neurons and glial cells from induced pluripotent stem cells (iPSCs) [25,26]. Transplantation of ENSCs in mouse models of congenital ENS disorders shows their ability to migrate and colonize mammalian guts [22,24,27]. However, so far, such stem cell-based therapeutic approaches have not been translated into the clinic, and practical limitations remain [27]. As this review focuses on ENS regeneration, an in-depth discussion of stem cell-based therapies is out of its scope. Additional information on ENSCs, their origins, in vitro/in vivo experiments, and their therapeutic potential can be found in several excellent recent reviews [15,24,27,28,29].

The other avenue for treating insufficient or injured ENS neurons in patients is to stimulate resident stem cells to regenerate the missing/damaged enteric neurons. Regeneration based on the activation of tissue-resident stem cells has been discussed as a promising approach for different organs, including bone, spinal cord, and retina [30,31,32,33]. For this approach to be successful in the ENS, a detailed understanding of the extent, potential, and cellular and molecular mechanisms underlying ENS regeneration is necessary. In this review, we will discuss the ability of the ENS to regenerate and why ENS regeneration might be limited in mammals. We will also discuss the high regenerative capacity of the ENS in the zebrafish model system and close with an outlook on the open questions and future directions for ENS regeneration research.

## 3. What Constitutes Nervous System Regeneration?

Nervous system regeneration is generally defined as either the repair or the new generation of neurons damaged by injury or disease. This can occur at two different levels: First, the regrowth of just neuronal axons when the neuronal cell body is not damaged. Second, “large scale” regeneration, where new neurons are generated and have to connect to the existing neural circuitry or build a new circuit that then is wired into the larger neural circuit [34]. Complete regeneration is viewed as the full restoration of lost neurons or neuronal function, whereas partial regeneration includes the generation of new neurons or nerve fibers without the complete restoration of lost neurons or neuronal function.

## 4. The Mammalian ENS Has Limited Regenerative Ability

Starting in the 1950s, different types of regeneration studies have been performed in the ENS in a variety of mammalian research organisms, including adult guinea pigs, rats, mice, and dogs [35,36,37,38,39,40,41,42,43,44,45,46,47,48,49,50,51,52,53,54,55,56,57]. Very few studies have so far been performed in non-mammalian species, for example zebrafish [58,59]. In mammals, neurons or nerve fibers are generated in some but not all experimental settings after injury (details of these experiments are found in the next sections). Generally, neuron numbers are not restored to control levels, and nerve fiber regeneration is often not complete. Additionally, within the injured area, significant structural changes remain. Several aspects might contribute to this variety in the regenerative responses: type of injury, observation time after injury, animal model, and which gut region has been injured. In the next section, we will discuss the main injury models used to study ENS regeneration and how the variability in experimental parameters might impact the regenerative response. The injury models that we discuss here are surgical or mechanical methods of injury, chemical-induced injury, infection with pathogens, and genetic models of Hirschsprung Disease.

## 5. ENS Regeneration after Surgical/Mechanical Injury in Mammals

**Figure 2:**
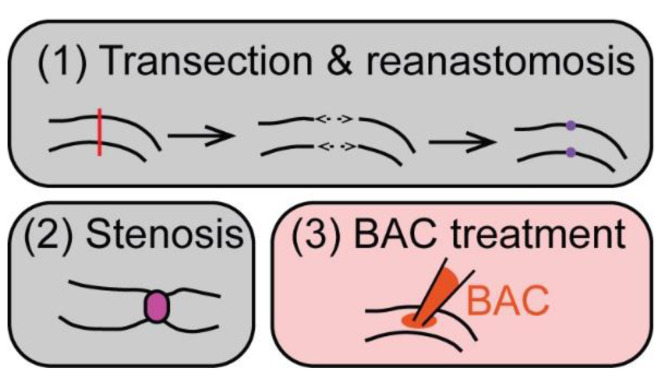
Common ENS injury approaches in mammals. Common models using surgical (grey boxes) or chemically induced (red) injury methods to study ENS regeneration in mammals: (**1**) transection and reanastomosis: the targeted part of the gut is transected (red line) with subsequent end-to-end anastomosis (purple). (**2**) For stenosis, a ring (magenta) is placed around the gut, which causes partial obstruction. (**3**) Benzalkonium chloride (BAC) treatment comprises the treatment of a small gut segment with BAC, which leads to a loss of ENS neurons in the treated area.

Transection and reanastomosis and (partial) stenosis are the main types of surgical injury that have been used to study ENS regeneration in various mammalian animal models (Figure 2).

Transection and reanastomosis in the small intestine in dogs, guinea pigs, or rats resulted in nerve fiber regeneration across the injury site with accompanying functional recovery. In dogs, transection and reanastomosis led to an initial loss of migratory motor complex (MMC) propagations after surgery [35,36]. MMCs are cyclic, sweeping gut movements in the small intestine during fasting [37]. After 40–60 days post-surgery, MMCs started to be coupled again between the two segments, indicating functional recovery of gut movements across the injury site. By approximately 100 days post-surgery, MMCs were fully recovered, suggesting regeneration of nerve fibers across the injury site [35,36].

Analysis of structural changes after surgical injury in the ENS in rats or guinea pigs showed that neurons degenerated in the injury site between 1 and 2 months after transection and reanastomosis in the small intestine [38,39,40]. At 6 weeks post-surgery, there were significantly fewer neurons within about 5 mm of the surgical site. Farther away, no difference in neuron numbers was seen. Even at 12 months, the surgical site did not contain any new neurons [38,39]. The area close to the surgical site showed an increase in large extra-ganglionic neurons forming clusters, but these additional neurons did not restore neuron numbers to control levels. A little farther away, in the area that had not seen a reduction in neuron numbers, large extra-ganglionic neurons emerged at 6 months and continued to increase in numbers until reaching a plateau at 1 year post-surgery [38]. These extra-ganglionic neurons were found in higher numbers than controls, forming clusters and connecting to surrounding enteric ganglia [38]. Interestingly, in mice, extra-ganglionic neurons that moved into enteric ganglia after 24 weeks were observed in the small intestine and colon after experimental treatment with a 5-Hydroxytryptamine receptor 4 (5-HT_4_) agonist to stimulate neurogenesis [41]. This indicates a potential connection between the process of adult neurogenesis and regeneration.

After 2–4 weeks, regenerating nerve fibers appeared in the lesion site both from the oral and aboral sides. These fibers extended further after 8 weeks, suggesting that nerve fiber regeneration across the lesion site also occurs in guinea pigs and rats [38]. Yet, structural changes in neuronal fiber patterns were visible even after 1 year post-surgery, indicating that nerve fibers were not fully restored across the lesion site [38]. Together, these studies showed that transection and reanastomosis in the small intestine led to distinct structural changes in neurons and nerve fibers depending on the distance from the surgical site and time after surgery, but never fully restore structural patterns as in the uninjured gut.

Analysis of structural changes after transection and reanastomosis in the lower GI tract in guinea pigs had contrasting results [42,43,44]. Surgery in the colon showed substantial and long-lasting disruption of neuron numbers and neuronal pathways at the lesion site [42]. Neurons degenerated at the injury site and failed to regenerate after 8–24 weeks. Nerve fiber regeneration occurred, but, in this model, regrowth of nerve fibers occurred preferentially in an oral to anal direction; no regrowth of fibers anal to oral was observed between 10 and 24 weeks after surgery [42]. In contrast, transection and reanastomosis in the guinea pig rectum led to nerve fiber regrowth across the lesion site accompanied by recovery of rectal contractions between 2 and 8 weeks [43,44]. In addition, new neurons were present in the injured site, but no ganglia were formed [43,44]. The difference in neuron and nerve fiber regeneration between the different gut regions suggests that each gut subdivision may have different abilities to restore neuronal function after surgical injury.

Injury models using (partial) stenosis focused on the small intestine [45,46,47,48,49]. Between 1 and 2 weeks after stenosis in rats, there was an increase in the numbers and cell volume of neurons per ganglia accompanied by thymidine incorporation or expression of the proliferation marker Proliferating Cell Nuclear Antigen (PCNA) in neurons upstream of the point of stenosis. This suggests that neurons either activated the cell cycle or were undergoing unscheduled DNA synthesis due to DNA repair [49]. It was not tested if the increase in neurons resulted in a full restoration of lost neurons. After 4 weeks, there was no evidence of proliferating cells, but the nuclear volume of neurons was still increased [45,46,47]. In a guinea pig model of stenosis, there was no evidence of an increase in neuron numbers after 3–5 weeks [48], but the earlier time points where cell cycle activation in neurons was observed in the rat models were not analyzed in the guinea pig model. Thus, parallel conclusions are unable to be made.

In summary, surgical injuries result in partial nerve fiber regeneration with functional recovery and some generation of neurons, but there is no full recovery of neuron numbers after surgery. There is some evidence that gut regions and species differ in their regenerative ability and response, but more extensive research needs to be performed in comparing the regenerative ability of different species and gut regions. Notably, regenerative processes take a long time, which is relevant for the experimental setup to study ENS regeneration.

## 6. ENS Regeneration after Chemically Induced Injury in Mammals

A chemically induced injury method commonly used to study ENS regeneration is treatment with benzalkonium chloride (BAC), which leads to a loss of ENS neurons in the treated area (Figure 2, [60,61]). In rat or mouse animal models, treatment with BAC of the small intestine or colon resulted in neuron degeneration and denervation within 2–5 days after treatment [50,51,52]. In the small intestine of rats, there was a significant increase in new neurons at the lesion site at 30–60 days, which suggests a partial regenerative response with ~25–30% of control neuron numbers present 60 days post-injury [52]. In contrast, no consistent generation of new neurons was observed in the treated mouse colon. Here, nerve fibers grew into the denervated area between 7 and 14 days post-treatment. Nerve fiber density increased until 60 days post-treatment to 35–60% of the control density. Occasionally, new neurons were present along the nerve fiber bundles, but neuron numbers did not increase significantly [51]. Genetic lineage tracing in the small intestine in mice also found that neuronal projections grew into the injured area along with newly generated enteric glial cells, but, at 3 months post-ablation, new neurons were not present within the injured area (though they were found adjacent to the area of injury) [50]. In the small intestine of rats, nerve fiber regrowth took even longer—at 15 days no neuron fibers were present. By 45 days, some neuronal fibers had regrown, but fibers showed marked structural differences to the control ENS [53].

In conclusion, injury of the ENS using chemical treatment results in denervation of the injured area. However, depending on the animal model and gut region, the timing of regrowth of nerve fibers and the regenerative ability differs. As with the surgical injury method, regeneration in the colon seems to be impaired compared with the small intestine, in which some limited regeneration of nerve fibers and ENS neurons was observed.

## 7. Regeneration in Animal Genetic Models of Hirschsprung Disease

A critical question for using regenerative approaches to treat ENS disorders is if, within the setting of the patient’s gut, progenitor cells can be stimulated to generate new ENS neurons. A recent study by Soret et al., (2020) tested if new neurons are generated in different genetic mouse models of Hirschsprung Disease after treatment of the distal gut with glial cell-derived neurotrophic factor (GDNF). GDNF treatment 4–8 days after birth resulted in the generation of new neurons and glial cells in the aganglionic colon, reaching on average 40% of wildtype neuronal density [62]. The newly-generated ENS ganglia conveyed functional recovery of gut motility, suggesting functional integration of the regenerated neurons [62]. Interestingly, the application of GDNF to adult guts in a chemical-induced injury model did not result in the generation of new ENS neurons in the ileum, pointing at variable responses to GDNF depending on the age or gut region [47]. GDNF treatment after birth provides a possible treatment approach for Hirschsprung Disease patients and might reduce the need for the surgical removal of the aganglionic gut region or result in better functional outcomes after surgery.

## 8. What Aspects Might Impact the Regenerative Process in Mammals?

The regeneration studies in mammals have shown that, even though there are instances of nerve fiber regeneration and partial regeneration of neurons, complete regeneration with restoration of neuron numbers to control levels does not take place. In this section, we will discuss three aspects that may impact the regenerative ability in the mammalian ENS.

### 8.1. Time Required for Full Regeneration

Regeneration events generally took a long time, and positive signs of regeneration were often not observed until months after injury. The reporting period of many studies was significantly shorter and may not cover a long enough time to determine if neuron regeneration might still happen. Thus, results determining limited or no regeneration capacity may be limited by the timeframe of the observation.

### 8.2. Impact of Inflammation

In the mammalian central nervous system (CNS), neuroinflammation is both detrimental and positive for a regenerative response. In zebrafish—a research organism with a remarkable ability to regenerate the nervous system post-injury—inflammatory responses support brain, retina, and spinal cord regeneration [63,64,65,66]. The gut contains a large number of immune cells, and intestinal inflammation results in structural changes in the ENS, including hyperplasia or loss of neurons [67,68]. Inflammatory responses have been evoked by different types of treatments, for example, with trinitrobenzene sulfonic acid (TSA), dextran sulfate sodium (DSS) [54,55], or by infection with the nematode *Nippostrongylus brasiliensis* [57]. Treatment with TSA resulted in an increase in proliferating cells at 3 and 7 d post-treatment, but the study did not investigate if this supported the generation of new neurons later [55]. DSS treatment in the colon led to a significant increase in the number of ENS neurons 2 days after treatment. In this treatment setting, a subset of enteric glial cells was suggested to give rise to ENS neurons [54,56]. Infection with *Nippostrongylus brasiliensis* resulted in a strong inflammatory response and led to a significant degeneration of nerve fiber densities until 21 days post-infection (dpi) [57]. Reinnervation peaked at 18 dpi, and experimental guts showed higher fiber density than in control animals [57]. Together, new neurons can be generated in an inflammatory setting. This suggests that inflammation might have positive effects on neural regeneration in the ENS. Even though inflammatory responses have been documented after ENS injury, many experimental settings/studies did not report if there was inflammation or not. In addition, it has not been directly tested if an inflammatory response impacts the regenerative processes as it does in the CNS. Thus, further study of the role of inflammation for ENS regeneration is necessary, specifically in the context of different types of injury.

### 8.3. Ability of Mammalian Adult Neurogenesis in the ENS

In the CNS, the ability to regenerate neurons in adults has been connected to the extent of adult neurogenesis; that is to say, high levels of adult neurogenesis correspond to the ability to regenerate the nervous system after injury. Low to no adult neurogenesis is generally correlated with low or no ability to regenerate neurons in the adult [64,69,70]. In the ENS, the extent of adult neurogenesis in mammals beyond postnatal stages has been debatedThe predominant view on neurogenesis in the mammalian ENS has been that the majority of enteric neurons are generated during embryogenesis and early postnatal stages with no continuous generation of neurons throughout adult life [41,47,50,54,71,72]. Only in specific experimental conditions, for example, induction of inflammation with DSS treatments or treatment with 5-HT_4_ agonists, has the generation of new neurons been observed in the adult [41,54]. Treatment with 5-HT_4_ agonists translates to improved neural regeneration, as nerve fiber regeneration and reflex recovery were accelerated in resection and anastomosis in the rectum of guinea pigs. In addition, the generation of new neurons after DSS treatment was abolished by treatment with a 5-HT_4_ antagonist [43,44,54]. This suggests that signals that support adult neurogenesis also promote neural regeneration in the ENS. The view of little to no adult neurogenesis in the ENS has been challenged in the study by Kulkarni et al., (2017) which observed high levels of adult neurogenesis in the gut with a steady turnover of ENS neurons in homeostatic conditions [73]. However, a new study by Virtanen et al., (2022) [72] did not find evidence for such robust, extensive ENS neurogenesis despite using the same experimental approach as [73]. Thus, this recent study supports the prevailing theory that there is little to no adult ENS neurogenesis under normal, physiological conditions [72]. Despite the lack of clarity regarding the extent of adult neurogenesis in vivo in the mammalian ENS, ENSCs can be isolated from the gut into adulthood and differentiate into ENS neurons in vitro [22,24]. This shows that stem cells that can generate new neurons are still present in the adult mammalian ENS, but work is still needed to de‘termine how these cells can be re-activated.

## 9. The Zebrafish ENS Regenerates Neurons after Focal Ablation

**Figure 3:**
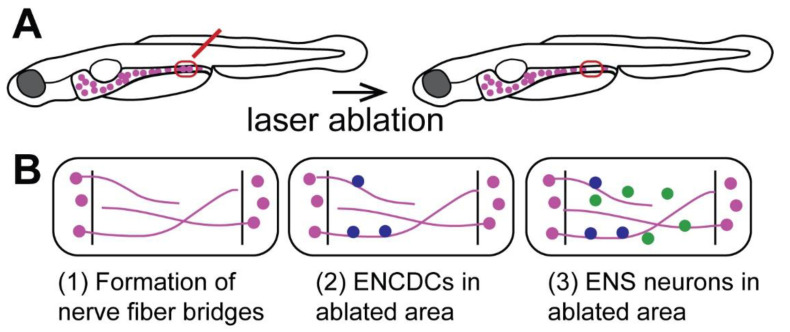
Regenerative processes in the zebrafish ENS after cell ablation. (**A**) Experimental setup of focal laser ablation (red) of a small set of ENS neurons in the zebrafish larvae. (**B**) Steps of regenerative processes in the zebrafish ENS in the ablated area marked by lines: (**1**) formation of nerve fiber bridge across the ablated area; (**2**) enteric neural crest-derived cells (ENCDCs, blue) appear in the ablated area; (**3**) new neurons (green) are generated in the ablated area [58].

Recent studies have found that zebrafish generate new neurons after focal ablation of a small fraction of ENS neurons (Figure 3A, [58,59]). This is consistent with the continuous generation of new ENS neurons into adulthood in zebrafish [74]. After laser-ablation of a small number of neurons in the distal zebrafish larval gut, new neurons are generated at the site of ablation, and neuron numbers increase 5 and 10 days post-ablation (dpa) at a significantly higher rate than in the control (Figure 3B). It remains to be determined if neuron numbers completely catch up to control levels and if these neurons restore gut function. The study by Ohno et al., (2021) also sheds light on potential cellular mechanisms that drive neuronal regeneration in the zebrafish ENS (Figure 3B). Nerve fibers first enter the ablated area at 1 dpa forming a bridge across the ablated area; enteric neural crest-derived cells (ENCDCs) also arrive via these nerve fiber bridges (Figure 3B). These ENCDCs proliferate, indicating that progenitor cell proliferation drives ENS regeneration in zebrafish [58]. These data suggest that zebrafish can generate new neurons after injury, but the occurrence of regeneration in adult zebrafish remains to be tested. Thus, zebrafish are a promising new research model to study the cellular and molecular mechanisms underlying ENS regeneration.

## 10. Outlook

Previous studies have shown that ENS regeneration in mammals is limited. Zebrafish have been put forward as a new model organism to study ENS regeneration. The zebrafish model has the potential to answer the questions of how a robust regeneration response is controlled on a cellular and molecular level. It will be important to determine if a more extensive loss of ENS neurons still results in robust regeneration in zebrafish. In addition, full functional recovery after neuron loss needs to be determined using approaches such as intestinal transit assay or gut motility imaging [75,76].

Important questions remain before regenerative approaches can be used to treat ENS disorders. The regenerative ability of mammalian models needs to be tested further with longer observation periods and a detailed analysis of functional recovery. This will also reveal important differences in the regenerative ability between ENS neuron types, gut regions, and species.

The field also needs a detailed understanding of how ENS regeneration is regulated on a cellular and molecular level in animal models with robust vs. limited regenerative capacity. This will allow for the determination of (1) which cell types are present during ENS regeneration, (2) which molecular changes take place, and (3) which cell lineages generate new neurons. For example, ENS glial cells generate neurons during adult ENS neurogenesis [74,77], and ENS glial cells have been suggested as a potential progenitor cell source in some injury settings [54,56]. In addition, Schwann cell precursors associated with extrinsic nerves have been identified as a source of new neurons after GDNF treatment in Hirschsprung Disease mouse models [62] and in zebrafish [59]. The regulatory programs underlying ENS regeneration also need to be determined. This information will provide the critical framework of cell biological processes and molecular-genetic factors essential for successful regeneration in the ENS. These are necessary steps for identifying differences in the cellular and molecular composition between model systems with high vs. low regenerative capacity in the ENS. Understanding these aspects of ENS regeneration will pave the way for using regenerative processes as a therapeutic approach to treating ENS disorders.
